# A Presentation of Necrotizing Disseminated Sporotrichosis

**DOI:** 10.7759/cureus.103906

**Published:** 2026-02-19

**Authors:** Phebie Rossi, Emily Eisenbraun, Oluwafunke O Ogunremi, John Looby

**Affiliations:** 1 Medical School, University of South Dakota Sanford School of Medicine, Vermillion, USA; 2 Department of Internal Medicine, Monument Health Rapid City Hospital, Rapid City, USA

**Keywords:** corticosteroid therapy, disseminated sporotrichosis, immunocompetent host, necrotizing cutaneous lesions, sporothrix schenckii

## Abstract

We report a biopsy-proven case of disseminated sporotrichosis in a 42-year-old immunocompetent male, highlighting an uncommon presentation with rapid clinical deterioration. The patient initially presented with cutaneous lesions involving the face, thorax, and extremities that were suspected to represent a delayed hypersensitivity reaction to trimethoprim-sulfamethoxazole, and he was treated with systemic corticosteroids. He returned 10 days later with worsening necrotic lesions and constitutional symptoms. Otolaryngology and dermatology services obtained multiple skin biopsies, which demonstrated numerous fungal yeast forms across distinct anatomic sites. Subsequent history revealed facial trauma from a tree branch while gardening several weeks before presentation. Plasma next-generation sequencing (Karius Spectrum Test) demonstrated strong molecular evidence of disseminated *Sporothrix schenckii*. Sporotrichosis is a subcutaneous mycosis typically acquired via traumatic inoculation and most commonly presents as localized disease in immunocompetent hosts; disseminated infection is rare and usually associated with immunosuppression. This case underscores the diagnostic challenge of sporotrichosis, the potential for corticosteroid exposure to exacerbate fungal infections, and the importance of early consideration of endemic mycoses in patients with progressive cutaneous lesions and relevant environmental exposures. The patient required intensive care for septic shock secondary to disseminated sporotrichosis and was treated with broad-spectrum antimicrobial and antifungal therapy.

## Introduction

Sporotrichosis is an infection with the dimorphic fungus *Sporothrix schenckii*, which exists in mold and yeast forms, that is typically acquired via traumatic inoculation or inhalation of spores [1]. *Sporothrix schenckii* is a saprophyte that is spread to humans primarily through soil, wood, or grain traumatic skin penetration, but has been reported zoonotically via scratch or bite from cats, armadillos, and rodents [1-3]. It is found worldwide, but is endemic to Central America, South America, and Africa [4]. Sporotrichosis classification was first described in 1959 as four categories, including lymphocutaneous, fixed cutaneous, extracutaneous, and disseminated [5]. Lymphocutaneous sporotrichosis is the most common form, presenting as ulcerated papules and ascending lymphangitis with mild or absent systemic symptoms [1,6]. Fixed cutaneous sporotrichosis presents as lesions restricted to the inoculation site without ascending lymphangitis [1,6]. Extracutaneous sporotrichosis is the rarest form, presenting as *Sporothrix schenckii* infection in the lungs, meninges, or skeleton without skin lesions [7]. Disseminated sporotrichosis is rare, often occurring in the setting of immunodeficiency, and affects the skin, lungs, meninges, and skeleton [1]. Disseminated sporotrichosis most often manifests in immunocompromised hosts in the setting of HIV/AIDS, diabetes mellitus, congenital immunodeficiencies, or immunosuppressive medications, but has been reported in immunocompetent hosts [8-10].

## Case presentation

**Figure 1:**
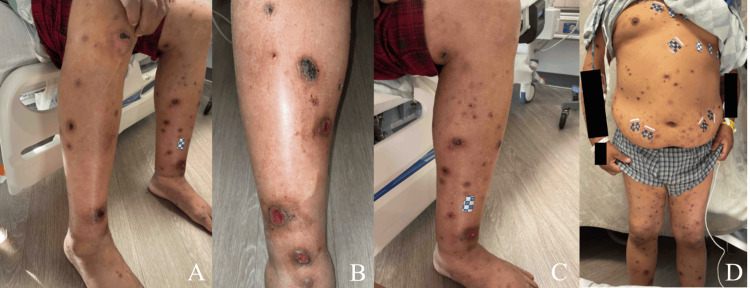
Clinical photographs on day two of first admission. Clinical photographs captured during the second day of the first hospitalization. (A) Scabbed lesions involving the bilateral lower extremities. (B) Scabbed and ulcerated lesions on the right lower extremity. (C) Scabbed and ulcerated lesions on the left lower extremity. (D) Lesions covering the thorax, abdomen, and bilateral lower extremities.

**Figure 2:**
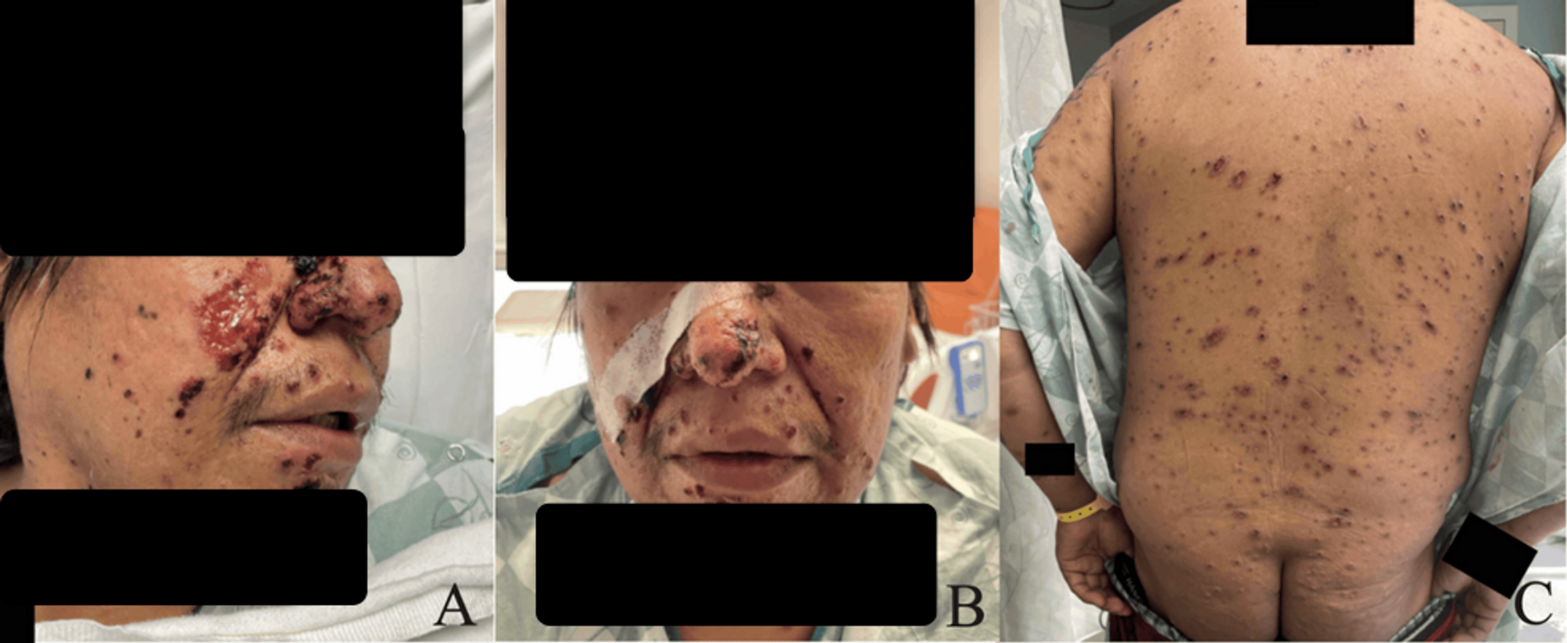
Clinical photographs on day two of the first hospitalization. Clinical photographs of the face and back captured during the second day of the first hospitalization. (A) Ulcerated lesion overlying the nasolabial fold with scabbed lesions present across the right nose and face. (B) Frontal view illustrating the asymmetric scabbed lesions across the patient’s face. (C) Scabbed lesions overlying the entirety of the patient’s back and upper buttocks.

A 42-year-old male initially presented to the emergency department for a diffuse rash with circular and ovoid scabbed lesions involving the face, anterior and posterior trunk, and all extremities (Figures 1, 2). This rash had been present for at least one month, per his account. He stated that he believed these began as bedbug bites that later became infected. The patient was evaluated by the Department of Infectious Diseases and had a negative workup for a variety of bacterial, viral, and fungal causes (Table 1). The reduced Protein C activity was notable at 21%, but the patient had no history of a chronic or hypercoagulable state. As such, it is unlikely to explain the initial susceptibility to disseminated sporotrichosis. Fungal culture on Sabouraud agar with potassium hydroxide stain was completed, and no fungal elements were seen. Two sets of blood cultures were drawn, and there was no growth at 120 hours in either set. A skin biopsy from the left leg was taken and demonstrated perivascular mixed inflammation with enlarged endothelial cells, and a central dilated hair follicle with chronic inflammation.

**Table 1:** Infectious diseases workup: part one. MRSA = methicillin-resistant *Staphylococcus aureus*; PCR = polymerase chain reaction

Laboratory test	Result	Reference range
Chlamydia RNA	Negative	Negative
*Neisseria gonorrhoeae* RNA	Negative	Negative
Hepatitis B surface antibody	<3.0 mIU/mL	Immune at >10 mIU/mL
Hepatitis B core IgM	Non-reactive	Non-reactive
Hepatitis C antibody	Non-reactive	Non-reactive
Syphilis treponemal antibody	Negative	Negative
HIV 1 antibody	Non-reactive	Non-reactive
HIV 2 antibody	Non-reactive	Non-reactive
P24 antigen	Non-reactive	Non-reactive
Varicella IgG antibody Index	2.9	Vaccinated: positive (> 1.1)
Varicella-zoster antibody, IgG	Positive	Negative
Varicella-zoster antibody, IgM	Negative	Negative
MRSA PCR	Negative	Negative
*Histoplasma *antigen	Not detected	Not detected
*Blastomyces* antigen	Not detected	Not detected
*Cryptococcus* antigen	Negative	Negative
Myeloperoxidase antibody	<0.2 U	<0.4 U
Protein C activity	21%	70–150%
Protein S activity	77%	65–150%
Proteinase 3 antibody (PR3)	<0.2 U	<0.4 U
Antinuclear antibody, HEp-2 Substrate	<1:80	<1:80

**Figure 3:**
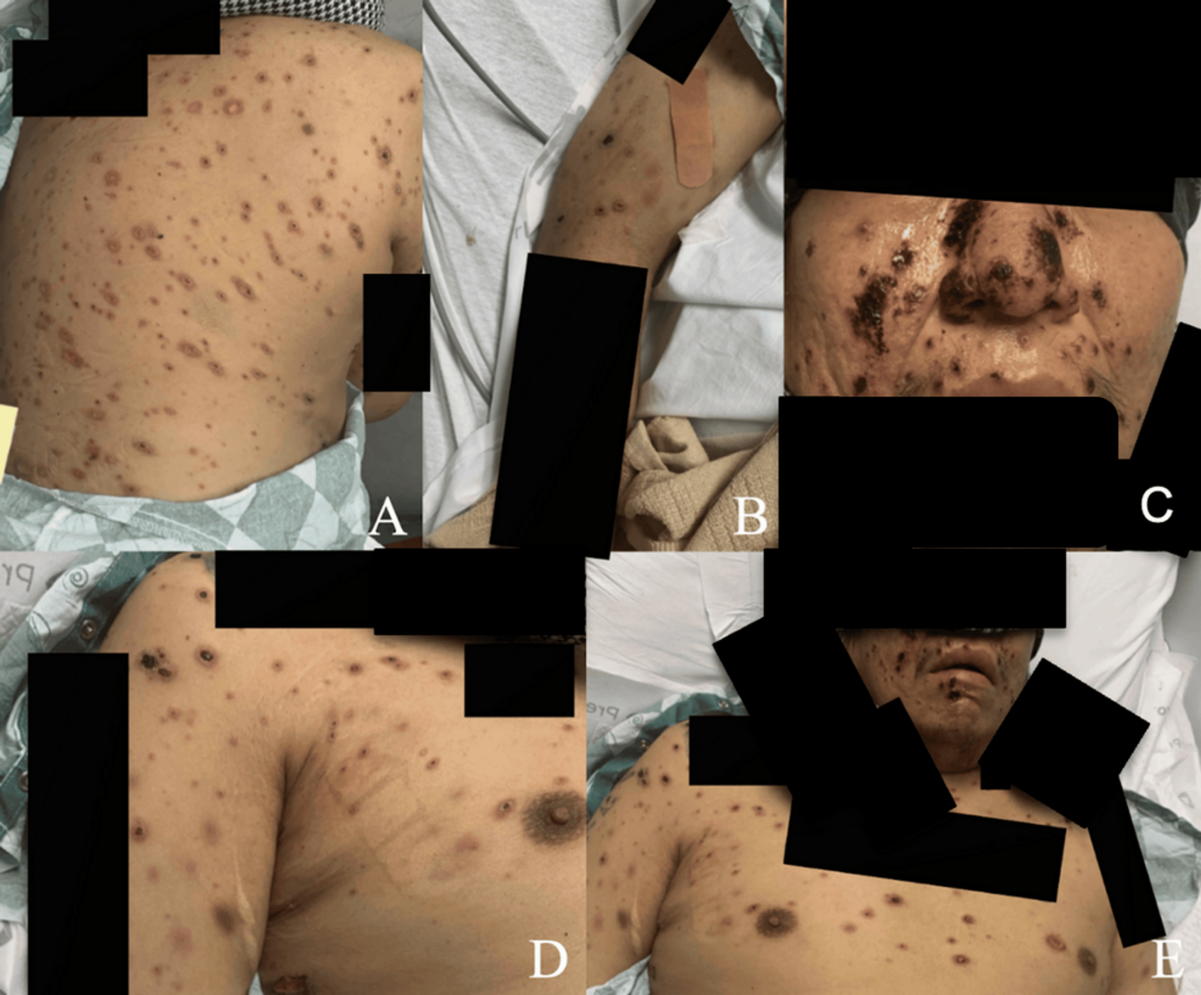
Clinical photographs on day seven of the first hospitalization. Clinical photographs captured during the seventh day of the first hospitalization. (A) Scabbed lesions overlying the posterior right shoulder and back. (B) Scabbed lesions overlying the right arm. (C) Scabbed lesions overlying the lower face. (D) Scabbed lesions overlying the right anterior shoulder and thorax. (E) Scabbed lesions spread across the lower face, right arm, and bilateral thorax.

**Figure 4:**
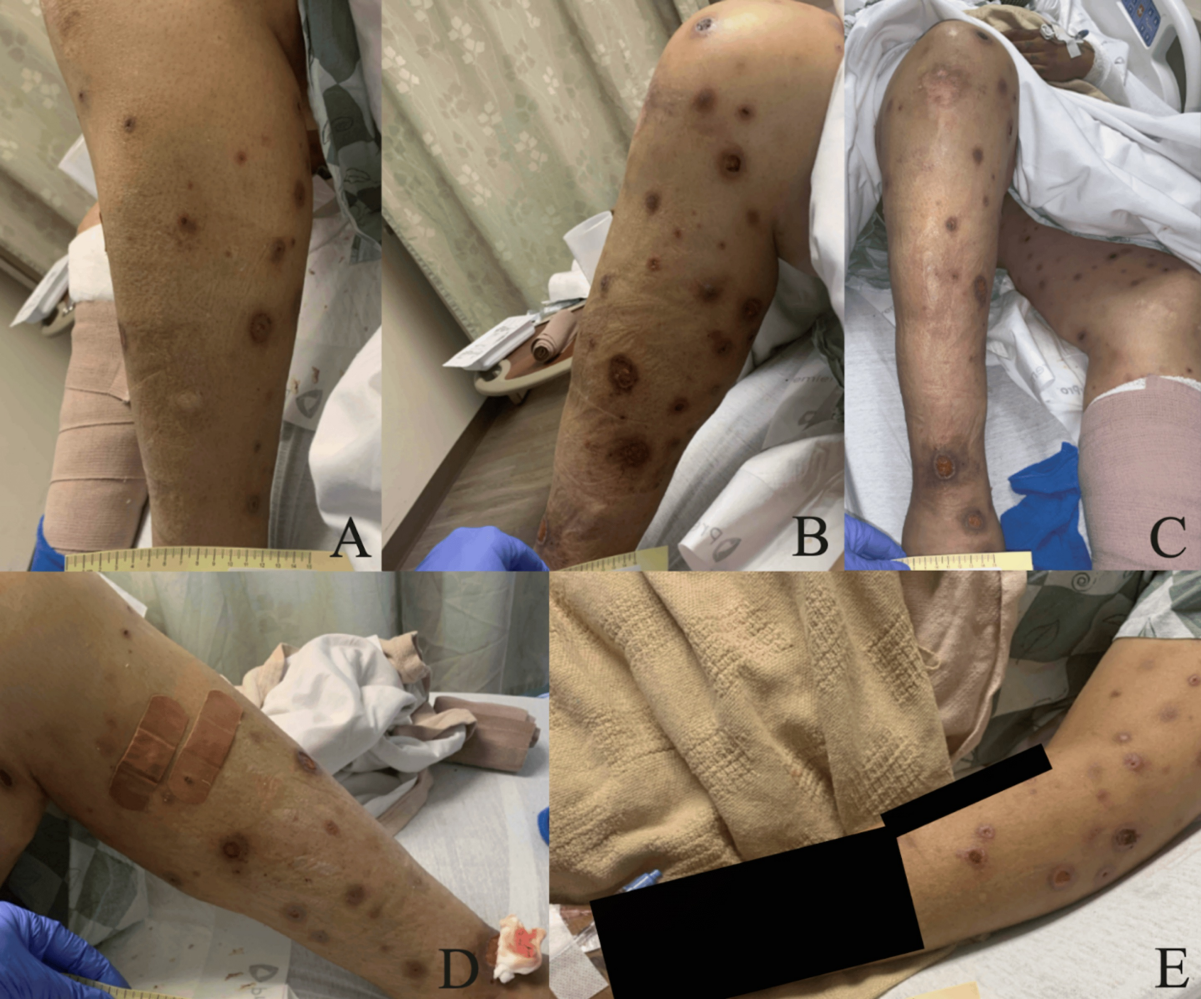
Clinical photographs on day seven of the first hospitalization. Clinical photographs captured during the seventh day of the first hospitalization. (A) Scabbed lesions on the left lateral leg. (B, C) Ulcerated lesions on the right lower leg. (D) Ulcerated lesions on the left medial leg. (E) Scabbed and ulcerated lesions on the left arm.

It was suspected at this time that the patient was experiencing an unusual, delayed drug reaction to trimethoprim-sulfamethoxazole, previously prescribed for soft-tissue infection at an outside facility. The patient was discharged on a prednisone taper for eight weeks, beginning at a dose of 60 mg for two weeks, followed by a 20 mg decrease every two weeks. The ulcerated lesions on his face scabbed over (Figure 3). Additional clinical photographs were captured depicting the lesions present on the arms and legs at the time of the patient’s discharge eight days after admission (Figure 4). During this first hospitalization course, the patient had been on vancomycin for a three-day course and ceftriaxone for a five-day course, with the first three days of the antimicrobials overlapping.

**Figure 5:**
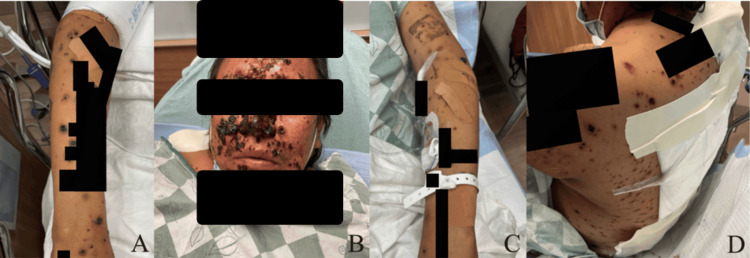
Clinical photographs on day three of the second hospitalization. Clinical photographs captured on the third day of the second admission. (A) Scabbed lesions on the right arm. (B) Erythematous papules and plaques with necrosis covering the central face. (C) Scabbed lesions present on the left arm. (D) Scabbed lesions on the left back.

**Figure 6:**
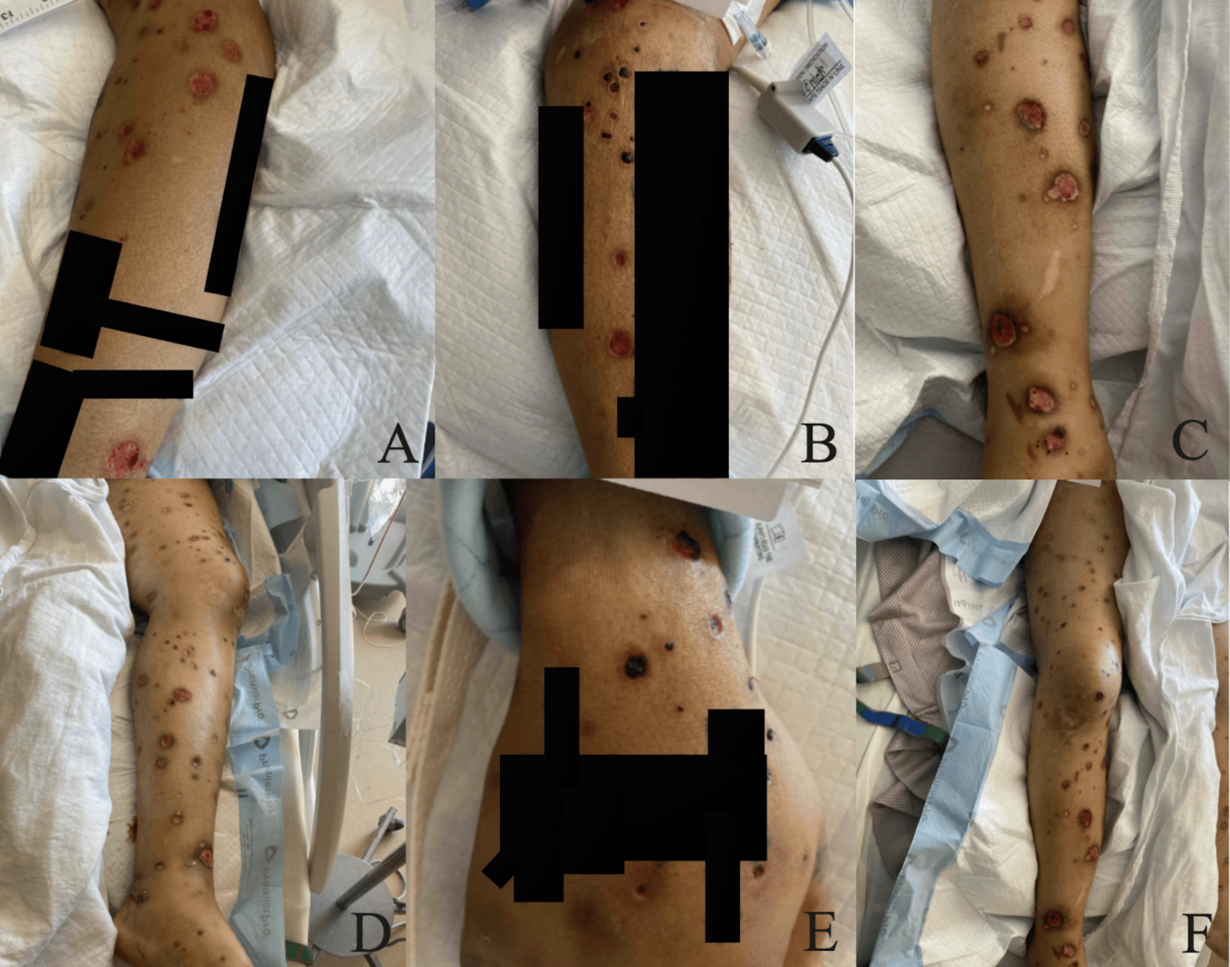
Clinical photographs on week two of the second hospitalization. Clinical photographs captured during the second week of the second admission. (A) Well-circumscribed ulcerated lesions overlying the left arm. (B) Scabbed and ulcerated lesions present on the right arm. (C) Ulcerated lesions overlying the right anterior shin. (D) Scabbed and ulcerating lesions present across the entirety of the left leg. (E) Scabbed and ulcerated lesions on the right hand. (F) Ulcerated and scabbed lesions on the entirety of the right leg.

Ten days after discharge, while on the 40 mg daily dose of the prednisone taper, the patient returned to the emergency department due to worsening of the rash and significant skin ulceration. Physical examination demonstrated diffusely painful ulcerative and necrotic lesions over the anterior and posterior thorax, arms, and legs. There were also erythematous papules and plaques with necrosis present over the central face (Figure 5). The rash did not involve the oral cavity, hands, or feet. He was also experiencing self-reported fever, chills, and rigors at this time. The following departments were consulted: Infectious Diseases, Dermatology, and Ear, Nose, and Throat (ENT). Biopsies were collected by both Dermatology and ENT (Tables 2, 3). Amphotericin B liposome was started by the Department of Infectious Diseases as further workup was completed. It was at this time that the patient stated a tree branch had hit him in the face several weeks prior while gardening (Table 4). Interfaced tests were sent to an external facility at this time. A Karius Spectrum Test was collected two days into his admission and sent as an interfaced test; results were available four days later. The Karius Spectrum Test demonstrated the presence of *Sporothrix schenckii* at a concentration of 8,869 molecules/100 nL. Treatment was adjusted appropriately with the goal of optimizing antifungal coverage while minimizing side effects, following existing recommendations favoring amphotericin B induction followed by prolonged azole therapy in disseminated disease (Table 5) [11]. The patient’s condition progressed, with deepening ulcerating lesions present on the bilateral lower legs (Figure 6). Eventually, he was transferred to the intensive care unit (ICU), as septic shock due to disseminated sporotrichosis and fungemia developed. After a course of several weeks in the ICU, the patient unfortunately passed away.

**Table 2:** Cutaneous and sinus biopsy results. GMS = Gomori methenamine silver; AFB = acid-fast bacilli

Location	Collection method	Microscopic description	GMS stain	AFB stain	Culture
Right dorsal forearm	Punch biopsy	Acute, chronic, and granulomatous dermal inflammatory infiltrate	Positive	Negative	N/A
Right inferior frontal hairline	Punch biopsy	Ulceration with acute, chronic, and granulomatous inflammatory infiltrate	Positive	Negative	N/A
Left sinus	Curettage	Sinonasal mucosa and necroinflammatory infiltrate debris involved by fungal yeast forms	Positive	N/A	Many (>50 colonies) *Staphylococcus aureus*
					Few (<50 colonies) *Serratia marcescens*

**Table 3:** Direct immunofluorescence results.

Laboratory test	Result
IgG	Negative
IgG4	Negative
IgM	Discontinuous granular basement membrane zone
IgA	Negative
C3	Focal granular basement membrane zone
Fibrinogen	Patchy staining of connective tissue fibers

**Table 4:** Infectious diseases workup during the second admission. PCR = polymerase chain reaction

Laboratory test	Result	Reference range
Procalcitonin (BRAHMS)	8.11 ng/mL	<0.50 ng/mL
Procalcitonin delta from peak	<0%	N/A
Aspergillus antigen, serum	<0.500 index	<0.500 index
Fungitell quantitative value	>500 pg/mL	<60 pg/mL
Fungitell qualitative result	Positive	Negative
Anaerobic culture	No anaerobic organisms isolated	No anaerobic organisms isolated
Lesion culture (right nasal sidewall)	Many (>50 colonies) *Serratia marcescens*	No colonies
*Coccidioides* antibody	Reactive	Negative
*Cryptococcus* antigen	Negative	Negative
Varicella-zoster virus PCR	Negative	Negative

**Table 5:** Antimicrobials over two hospitalization courses.

Dates of administration	Medication
6/20–6/21	Vancomycin
6/20–6/24	Ceftriaxone
7/8–7/10	Amphotericin B liposome
7/8–7/10	Piperacillin-tazobactam
7/8–7/9	Acyclovir
7/8–7/10	Posaconazole
7/10–7/13	Ceftaroline
7/12–7/16	Amphotericin B liposome
7/10–7/16	Itraconazole
7/17	Linezolid
7/17	Piperacillin-tazobactam
7/17	Isavuconazonium

## Discussion

Sporotrichosis, caused by *Sporothrix schenckii*, may present in one of three cutaneous forms, namely lymphocutaneous, fixed, or disseminated, which can notably present cutaneously or systemically [7]. The lymphocutaneous presentation is most frequently seen in immunocompetent patients, presenting as ulcerated nodules on the limbs and face [7]. Data suggest that the lymphocutaneous presentation is the most prevalent, seen between 70-75% of the time, while the cutaneous presentation is seen 20-30%, and the disseminated presentation is seen 5% of the time [12].

The disseminated form of sporotrichosis can present in a variety of ways, as cases have described findings on all surfaces, including mucous membranes, bones, joints, and the central nervous system [12]. Disseminated sporotrichosis is typically associated with an immunocompromised status, yet several published cases describe immunocompetent hosts developing this form of the disease. One such case presented an immunocompetent male with nodular plaques across his chest, limbs, and abdomen who responded favorably to treatment with potassium iodide [13].

Our patient’s initial presentation mimicked a drug-induced hypersensitivity reaction or vasculitis, leading to corticosteroid therapy that likely exacerbated fungal dissemination. It is well known that glucocorticoids suppress both the innate and adaptive immune system, but there is a lack of research noting the incidence of disseminated sporotrichosis following steroid use [14]. Cases have been presented in which patients were misdiagnosed initially, received immunosuppressive agents including steroids, and then presented with cutaneous disseminated sporotrichosis [15]. Yet, further information, including the length of time or the dosing of the steroid, was not discussed. As such, the clinical course highlights a potential pitfall in management, as empiric corticosteroid use without definitive exclusion of infection may have worsened this patient’s outcome.

Disseminated sporotrichosis poses a diagnostic challenge due to its varied clinical presentation. As shown in Figures 1-6, this patient presented with lesions on his face, trunk, and limbs that eventually developed into shallow ulcers. There was no apparent bone, joint, or mucosal involvement in this case. Data suggest that an initial misdiagnosis is not rare and that the diagnosis is frequently delayed due to a varied presentation that mimics other inflammatory conditions [16]. Additional barriers included this patient’s delayed disclosure of a branch scratch, highlighting the clinical significance of both repeated and detailed histories that include pointed questioning of environmental and occupational exposures. Such variability underscores the importance of maintaining a broad differential in patients with necrotic skin lesions and systemic symptoms, particularly when initial cultures are unrevealing.

Plasma microbial cell-free DNA (cfDNA) sequencing has emerged as a valuable adjunctive diagnostic tool in the evaluation of suspected invasive fungal infections, particularly when conventional cultures and histopathology are nondiagnostic or delayed. In a large clinical cohort of over 15,000 patients, Park et al. demonstrated that plasma cfDNA next-generation sequencing could identify a broad spectrum of pathogens, including invasive fungal organisms, from a single non-invasive blood sample [17]. This approach offers several advantages in critically ill patients, including the ability to detect disseminated infections without reliance on tissue acquisition, which may be limited by sampling error, prior antimicrobial exposure, or procedural risk. Importantly, cfDNA sequencing can provide earlier pathogen identification compared with traditional culture-based methods, potentially enabling more timely initiation of targeted antifungal therapy. In complex cases such as this one, where initial biopsies and cultures were unrevealing despite progressive disease, plasma cfDNA testing can play a pivotal role in establishing the diagnosis and guiding management, underscoring its growing clinical relevance in invasive fungal disease diagnostics.

Management of disseminated sporotrichosis requires prompt initiation of antifungal therapy, namely, with amphotericin B [18]. After the patient stabilizes, itraconazole is recommended as it is known to be effective and safe [18]. Historically, potassium iodide was used in treatment and can still be utilized today in patients unwilling or unable to use amphotericin B [13]. Many studies illustrate a high rate of mortality associated with disseminated sporotrichosis, with one suggesting that sporotrichosis-related deaths occurred in 42.9% of their patients [19]. While informative, this data cannot fully explain our patient’s presentation as he did not have any identifiable underlying immunodeficiency, and the studies published involved only small numbers of cases. As such, this case highlights that disseminated sporotrichosis exists on a spectrum of severity, even among immunocompetent hosts, and reinforces the importance of early recognition and aggressive antifungal therapy to optimize outcomes.

## Conclusions

This case underscores the diagnostic and therapeutic challenges associated with disseminated sporotrichosis, particularly in immunocompetent patients, where such presentations are rare and easily misattributed to other inflammatory or drug-induced conditions. The exacerbation of disease following corticosteroid therapy highlights a critical pitfall in management: the initiation of immunosuppressive treatment before infection is definitively excluded may contribute to fungal dissemination and worse outcomes. While disseminated sporotrichosis is classically linked to immunocompromised states, case reports, including this case, illustrate its potential to affect otherwise healthy patients, further broadening the clinical spectrum of disease. Ultimately, this case reinforces the necessity of maintaining a broad differential diagnosis in patients with necrotic skin lesions and systemic symptoms, pursuing timely diagnostic evaluation, and promptly initiating antifungal therapy. Early recognition and appropriate management remain pivotal in reducing morbidity and mortality associated with disseminated sporotrichosis.
